# Multivariate analyses in microbial ecology

**DOI:** 10.1111/j.1574-6941.2007.00375.x

**Published:** 2007-09-25

**Authors:** Alban Ramette

**Affiliations:** Microbial habitat group, Max Planck Institute for Marine Microbiology Bremen, Germany

**Keywords:** ordination, multivariate, modeling, statistics, gradient

## Abstract

Environmental microbiology is undergoing a dramatic revolution due to the increasing accumulation of biological information and contextual environmental parameters. This will not only enable a better identification of diversity patterns, but will also shed more light on the associated environmental conditions, spatial locations, and seasonal fluctuations, which could explain such patterns. Complex ecological questions may now be addressed using multivariate statistical analyses, which represent a vast potential of techniques that are still underexploited. Here, well-established exploratory and hypothesis-driven approaches are reviewed, so as to foster their addition to the microbial ecologist toolbox. Because such tools aim at reducing data set complexity, at identifying major patterns and putative causal factors, they will certainly find many applications in microbial ecology.

## Introduction

Microbial ecology is undergoing a profound change because structure–function relationships between communities and their environment are starting to be investigated at the field, regional, and even continental scales (e.g. Hughes Martiny *et al*., 2006; Ramette & Tiedje, 2007a, b). Because DNA sequences are being accumulated at an unprecedented rate due to high-throughput technologies such as pyrosequencing (Edwards *et al*., 2006a, b), single-cell genome sequencing (Zhang *et al*., 2006), or metagenomics (Venter *et al*., 2004; Field *et al*., 2006; Gill *et al*., 2006), future challenges will very likely consist of interpreting the observed diversity patterns as a function of contextual environmental parameters. This would help answer fundamental questions in microbial ecology such as whether microbial diversity responds qualitatively and quantitatively to the same factors as macroorganism diversity (Horner-Devine *et al*., 2004; van der Gast *et al*., 2005; Green & Bohannan, 2006; Hughes Martiny *et al*., 2006).

**Fig. 1:**
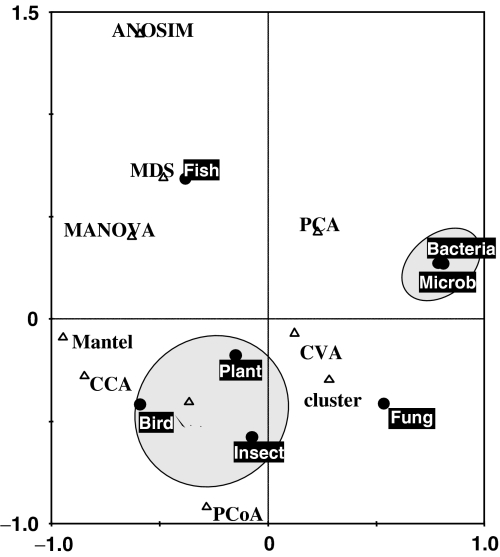
Correspondence analysis of method usage in various scientific fields. In this symmetrical scaling of CA scores, the first two axes explained 47.3% and 35.8% of the total inertia of Table 1, respectively. The gray areas were drawn to facilitate the interpretation. Complete row names (scientific fields; full circles) and column names (methods; white triangles) are given in Table 1. Methods (triangles) located close to each other correspond to methods often occurring together in studies. The distance between a scientific field point and a method point approximates the probability of method usage in the field.

Most obstacles encountered by microbial ecologists when they try to summarize and further explore large data sets concern the choice of the adequate numerical tools to further evaluate the data statistically and visually. Such tools, which have been developed by community ecologists to work on distribution and diversity patterns of plants and animals, could be readily applied in microbial ecology. Although multivariate analyses of community diversity patterns are well described in the literature, microbial ecologists have used multivariate analyses either rarely or mostly for exploratory purposes. A brief survey of the literature confirms this trend (Table 1; Fig. 1). Table 1 indicates that bacterial studies rank third after plant and fish studies for their use of multivariate analyses. Complex data sets are mostly explored via principal component analysis, or cluster analysis, and hypothesis-driven techniques such as redundancy analysis, canonical correspondence analysis (CCA), or Mantel tests are more rarely used (Fig. 1). Axis 1 (horizontal) clearly differentiates microscopic (bacteria, microorganisms, fungi) from macroscopic (fish, bird, plant, insect) life, and this may be related to the use of more exploratory methods (e.g. cluster analysis, PCA) in the first group. It is important to state that the figures presented in Table 1 and Fig. 1 have to be taken with caution because many articles do not include a description of statistical approaches in their titles or abstracts, and so the table is certainly biased and incomplete. However, the point of the table was both to identify some general trends in the literature and to give one example of the usefulness of multivariate analysis to analyze a data table.

**Table 1:** Usage (%) of multivariate methods in different fields

	Exploratory analysis				Hypothesis-driven analysis						
			
Keywords†	Cluster	PCA	MDS	PCoA	CCA	RDA	manova	Mantel	anosim	CVA	Total number‡
Bacter^*^	48.5	38	4.5	0.4	3.2	1.8	1.3	0.4	0.9	1.1	1141
Microb^*^	45.8	40.2	3.9	1.1	2.2	2.2	1.1	1.7	0.6	1.1	179
Plant^*^	40.3	28.5	4.6	1.7	15.5	3.7	1.9	2.3	0.6	0.9	3335
Fung^*^	54	27.2	2.8	1.1	8.5	2.8	0.9	1.1	0.2	1.4	563
Fish^*^	30.1	33.7	9.8	0.3	13.5	2.7	3.6	2.9	2.3	1.2	1464
Bird^*^	41	20.5	5.4	0.7	21.2	3.5	2.1	4.2	0.5	0.9	429
Insect^*^	54.3	13.7	6.1	0.8	11.5	4.4	3.5	3	1.1	1.7	637

A literature search was performed with the Thomson ISI research tool with the following parameters (Doc type, all document types; language, all languages; databases, SCI-EXPANDED, SSCI, A&HCI; Timespan, 1900–2006) on December 13, 2006 in the titles and abstracts of the articles only.

† Asterisks were placed at the end of each keyword to accommodate for variations. Each keyword was additionally combined with the following technical designations: cluster, cluster analysis; PCA, principal component analysis; MDS, multidimensional scaling; PcoA, principal coordinate analysis; CCA, canonical correspondence analysis; RDA, redundancy analysis; Mantel, Mantel test, or CVA, canonical variate analysis.

‡ Total number refers to the total number of publications identified by each keyword and all its combinations. The ordination based on correspondence analysis of the raw number is depicted in Fig. 1.

This review aims at presenting some common multivariate techniques in order to foster their integration into the microbial ecologist's toolbox. Indeed, ‘it is no longer possible to gain a full understanding of Ecology and Systematics without some knowledge of multivariate analysis. Or, contrariwise, misunderstanding of the methods can inhibit advancement of the science’ (James & McCulloch, 1990). Such a review is ambitious because it tries to provide a few guidelines for a very vast discipline that is still under development. For this reason, it cannot be exhaustive and does not pretend to offer in-depth coverage of all selected topics. The review is largely inspired by descriptions, comments, and suggestions originating from multiple, highly recommended sources (ter Braak & Prentice, 1988; James & McCulloch, 1990; Legendre & Legendre, 1998; Leps & Smilauer, 1999; ter Braak & Smilauer, 2002; Palmer, 2006), where detailed information about each technique can be obtained.

In the first part, data type and preparation are reviewed as a necessary basis for subsequent multivariate analyses. Second, common multivariate methods (i.e. cluster analysis, principal component analysis, correspondence analysis, multidimensional scaling) and a few statistical methods to test for significant differences between groups or clusters are described, focusing on the methods' main objectives, applications, and limitations. Beyond the mere identification of diversity patterns, microbial ecologists may wish to correlate or explain those patterns using measured environmental parameters, and this approach is addressed in the third part. Special emphasis is placed on a few methods that have proven useful in ecological studies, namely redundancy analysis, CCA, linear discriminant analysis, as well as variation partitioning. The final part provides practical considerations to help researchers avoid pitfalls and choose the most appropriate methods.

## Data types and data preparation

### Data sets

The initial multivariate data set may consist of a table of objects (e.g. samples, sites, time periods) in rows and measured variables for those objects in columns. This table structure is the standard used in the present review. When the latter variables are biological taxa, the columns will simply be designated as ‘species’ thereafter. It is critical to clearly identify what corresponds to objects and variables in the data set. Indeed, objects in one study may be species or operational taxonomic units (OTU) for which catabolic profiles, gene presence or polymorphism, etc. are measured. In another study where samples from different sites are compared based on, for instance, community fingerprinting techniques, objects can now be samples and species variables. This distinction is important because procedures that analyze relationships among objects or among variables are different. Objects are defined *a priori* by the sampling strategy before making observations and variable measurements. Besides, most multivariate analyses assume independence between objects (or samples), i.e. observations made on an object are not *a priori* dependent on those made on another object. Variables, however, can be found to be intercorrelated to various degrees, but this is not necessarily known in advance. Initial data sets can also consist of distance matrices where pairwise dissimilarities between objects are calculated. The original table of raw data is not always available, e.g. for DNA–DNA hybridization values, phylogenetic distances, and thus specific multivariate techniques have to be considered to deal with data matrices.

### Data transformations

In multivariate data tables, measured variables can be binary, quantitative, qualitative, rank-ordered, classes, frequencies, or even a mixture of those types. If variables do not have a uniform scale (e.g. environmental parameters measured in different units or scales) or an adequate format, variables have to be transformed before performing further analyses. Each qualitative variable has to be recoded as a set of numerical variables that replace it in the numerical calculations. One way to do so is to create a series of ‘dummy’ variables that correspond to all the states of the qualitative variable. For instance, if the variable ‘season’ has to be recoded, four associated variables will be constructed, and for each object the value 1 will be given to the corresponding season when it occurs, and 0 for the three other seasons when it is absent. Many statistical packages automatically perform this recoding.

*Standardization* provides dimensionless variables and removes the undue influence of magnitude differences between scales or units. A common procedure is to apply the *z*-score transformation to the values of each variable. For each variable, it consists of (1) computing the difference between the original value and the mean of the variable (i.e. centering) and of (2) dividing this difference by the SD of the variable.

*Normalizing* transformations aim at correcting the distribution shapes of certain variables, which depart from normality. One thus tries to obtain a homogeneous variances for variables, conditions under which multivariate procedures often perform better. Different mathematical transformations can be used to normalize the *x* values of a variable: for instance, the arcsin (√*x*) transformation can be applied to percentages or proportions, log(*x*+*c*) to variables departing strongly from a normal distribution, and √(*x*+*c*) to less problematic cases, with *c* being a constant that is added to avoid mathematically undefined computations. The *c* constant is generally chosen so that the smallest nonzero value is obtained by computing *x*+*c* in the former functions. The constant should also be of the same order of magnitude as the variable (Legendre & Legendre, 1998).

To make community composition (either presence–absence or abundance) data containing many zeros suitable for analysis by linear methods such as principal component analysis (PCA) or canonical redundancy analysis (RDA), the Hellinger transformation [Eq. (1)] is one of five transformations that give good results (Legendre & Gallagher, 2001). The chord transformation is a useful transformation that also gives less weight to rare species in the species table [Eq. (2)]. The transformations are given by 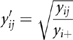
(1)
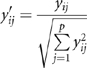
(2)where *y*_*ij*_ is the original species value for site *i* and species *j*, *y*_*i*+_ represents the sum of all species values for site *i* (i.e. sum per row), *p* is the number of species in the table (number of columns), and 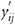 represents the resulting, transformed species value (Legendre & Gallagher, 2001). These transformations are particularly recommended when rare species are not truly rare, i.e. when they mostly occur because the sampling was performed blindly, as generally done in soil or marine microbial ecology. Further data transformations can be found in Sokal & Rohlf (1995) and Legendre & Legendre (1998).

The way to deal with missing data is a discipline on its own (Legendre & Legendre, 1998). Briefly, one can either delete rows or columns containing the missing value(s), or try to replace the missing values by mathematical estimates inferred from values obtained from other objects in the data set. In the latter case, it is still difficult to provide ecologically meaningful explanations for these estimates. In any case, the specific handling of missing data should be reported by the investigator.

When dealing with matrices, it is possible to change a similarity matrix (*S*) into a dissimilarity matrix (*D*) by applying the following transformations: *D*=1−*S*, *D*=√(1−*S*), or *D*=√(1−*S*^2^). To normalize any *D* matrix to the interval [0–1], one can compute *D*/*D*_max_, or (*D*−*D*_min_)/(*D*_max_−*D*_min_), where *D*_max_ and *D*_min_ represent the highest and lowest values of *D*, respectively (Legendre & Legendre, 1998).

## Exploratory analyses

### Visualization and exploration of complex data sets

The basic aim of ordination and cluster analysis is to represent the (dis)similarity between objects (e.g. samples, sites) based on values of multiple variables (columns) associated with them, so that similar objects are depicted near from each other and dissimilar objects are found further apart from each other. Exploratory multivariate analyses are thus useful to reveal patterns in large data sets, but they do not directly explain why those patterns exist. This latter point is addressed in the third part of the review.

#### Cluster analysis and association coefficients

Cluster analysis encompasses several multivariate techniques that are used to group objects into categories based on their dissimilarities. The aim is both to minimize within-group variation and maximize between-group variation in order to reveal well-defined categories of objects, and therefore reduce the dimensionality of the data set to a few groups of rows (James & McCulloch, 1990; Legendre & Legendre, 1998). This approach is thus generally recommended when distinct discontinuities instead of continuous differences (i.e. gradients) are expected between samples (objects) because cluster analysis mostly aims at representing partitions in a data set (Legendre & Legendre, 1998).

Because distance matrices that are based on differences in DNA or amino acid sequences are commonly used to describe microbial diversity, cluster analysis has become very popular in microbial ecology (Table 1; Fig. 1). This is not surprising because the grouping of organisms based on their phenotypic or genotypic similarities in order to infer their taxonomic positioning is generally and historically based on cluster analysis (or at least based on a tree-like representation) and, as such, is central to biology and evolution (Avise, 2006). Typical microbial ecology questions that are addressed by cluster analysis are whether the clustering patterns of molecular sequences reflect sample origin or sampling time in order to reveal specific biogeographical or temporal patterns, respectively (Whitaker *et al*., 2003; Acinas *et al*., 2004). Those factors are generally hypothesized to be of a discontinuous nature, but the rationale of generally representing molecular differences as discontinuous clusters in microbial ecology and microbial genomic studies has only started to be questioned (Konstantinidis *et al*., 2006). Another common application consists of sorting out clones from environmental samples based on specific criteria (e.g. genetic or phenotypic markers) because clones or variants are expected to form tight clusters around their parental strains and to be more distinct from other lineages (Acinas *et al*., 2004). In microarray data analysis, cluster analysis has helped identify common expression patterns of groups of genes, which may shed light on functionally related genes or pathways (Eisen *et al*., 1998).

Cluster analysis of a data table proceeds in two steps. First, a relevant association coefficient has to be chosen to measure the association (similarity or dissimilarity) among objects or among variables. Second, the calculated association matrix is represented as a horizontal tree (hierarchical clustering) or as distinct groups of objects (*k*-means clustering), based on specific rules to aggregate objects. For ecologists, the power of cluster analysis derives from the existence of different types of (dis)similarity coefficients. The choice of appropriate and ecologically meaningful association coefficients is particularly important because it directly affects the values that are subsequently used for the categorization of objects.

The analysis of similarities among objects (rows) is designated as *Q* mode analysis, whereas when relationships among variables (columns) are the focus of the study, this is referred to as *R* mode analysis (Legendre & Legendre, 1998). Noticeably, the two modes of analysis do not generally use the same association coefficients. Although it is not possible to give a full review of all association coefficients here, it is useful to known that, for comparing objects (rows) based on their column attributes in *Q* mode analysis, coefficients may be chosen as a function of data type (quantitative, qualitative, ordinal, or mixed data, normalized data, presence-absence), importance given to rare species, weight given to each object, and calculation of associated probability levels. For comparing objects in a sample-by-environment table (e.g. water, soil chemistry), selection of appropriate coefficients generally depends on data type and unit homogeneity of the measured variables. In *R* mode analyses, in addition to the previously cited criteria, the choice of a coefficient may also depend on how the variables are related to each other (e.g. linearly, monotonically, qualitatively, ordered), and on how species absence is handled in the calculations. In most ecological studies, the absence of a species at two sites being compared is not considered as a measure of similarity between those sites. Indeed, a simultaneous species absence at two sites may be due to different reasons, e.g. the sites offer different physical–chemical conditions and the species cannot exist under both conditions, and so there is no straightforward conclusion about site similarity that can be drawn in this case. *Asymmetric* coefficients are coefficients that do not take into account cases of double absences of species (‘double zeros’) in the calculation of pairwise similarities among sites. Moreover, in microbial ecology where environmental communities are generally far from being exhaustively sampled, a double absence of an OTU has to be regarded more as a lack of information rather than a sign of common structure among samples, and asymmetric coefficients such as Jaccard (1901) or Sørensen (1948) should be preferred. More details about the calculation of association coefficients and their appropriateness can be found, for instance, in Chapter 7 of (Legendre & Legendre, 1998).

When an association matrix is calculated, the relationships between objects or variables can be represented following specific aggregation rules. Three general approaches are commonly used: hierarchical clustering, *k*-means partitioning, and two-way joining. In *hierarchical clustering*, a linkage rule to form clusters and the numbers of clusters that best suit the data have to be determined *a priori*. Clusters, which are nested rather than mutually exclusive here, are either formed by progressively agglomerating objects from high to low similarity cutoff values (forward clustering), or using the converse strategy, i.e. grouping all cases together and progressing from low to high cutoff values in order to merge objects and clusters (backward clustering). These two strategies do not necessarily yield the same clusters. The merging of clusters is visualized using a tree format (generally horizontal) and is successful when well-defined clusters are identified in the data set (Sneath & Sokal, 1973).

Common linkage rules are, e.g. *nearest neighbor* (the distance between two clusters is the distance between their closest neighboring points), *furthest neighbor* (the distance between two clusters is the distance between their two furthest objects), and the widely used unweighted pair-group method using averages (UPGMA; Sneath & Sokal, 1973), where the distance between two clusters is the average distance between all intercluster pairs. When within-cluster homogeneity is desired, *Ward's method*, which merges clusters only if they increase the within-cluster variation the least, is recommended (Legendre & Legendre, 1998). Finally, equal weight can also be given to clusters that are expected to be of different sizes using the *weighted arithmetic average clustering* (WPGMA), which consists of giving less weight to the original similarities of the largest groups (Legendre & Legendre, 1998).

In *k-means clustering*, objects are assigned to *k* clusters (*k* being defined in advance), based on their nearest Euclidean distance to the mean of the clusters. The mean of the cluster is iteratively recalculated until no more assignments are made and cluster means fall below a predefined cut-off value or until the iteration limit is reached. Different means for each cluster are ideally obtained for each dimension used in the analysis, as indicated by high *F*-values from the respective analyses of variance. Unlike hierarchical clustering, *k*-means clustering does not require prior computation of dissimilarity matrix among objects and is therefore more adapted to large data sets (e.g. few thousand objects) where computing power is an issue. However, the method is quite sensitive to outliers, which are usually removed before performing the analyses (Legendre & Legendre, 1998).

*Two-step cluster analysis* may be useful to group objects into clusters when one or more of the variables are categorical (not interval or dichotomous). Objects are first grouped based on the categories, which are themselves hierarchically clustered as single cases. Because neither a proximity table nor iterative steps are required, the method is particularly suited for the analysis of very large data sets (Eisen *et al*., 1998).

#### Principal component analysis (PCA)

PCA has been applied to numerous phenotypic and genotypic (e.g. fingerprinting patterns) data sets, and it is one of the most popular exploratory analyses (Table 1), perhaps because the technique is generally the first multivariate approach to be explained in most data analysis manuals. However, this choice may not always be justified in ecology and recommendations for appropriate applications are provided at the end of this section and in the ‘Practical considerations’ part of the present review. Examples of use in microbial ecology concern the identification of patterns of microbial community change over seasons or geographic areas (e.g. Merrill & Halverson, 2002), or as those patterns relate to different plant compartments at different plant developmental stages (Mougel *et al*., 2006), or the reduction of the complexity of data sets involving hydrochemistry data, bacterial, and archeal community profiles in order to visualize and interpret complex multivariate data sets onto two-dimensional geographic maps of contaminated sites (Mouser *et al*., 2005).

The PCA procedure basically calculates new synthetic variables (principal components), which are linear combinations of the original variables (for instance, the species of a sample-by-species table), and that account for as much of the variance of the original data as possible (Hotelling, 1933). The aim is to represent the objects (rows) and variables (columns) of the data set in a new system of coordinates (generally on two or three axes or dimensions) where the maximum amount of variation from the original data set can be depicted. In practice, PCA is either performed on a *variance–covariance* matrix or on a *correlation matrix*. The first approach is followed when the same units or data types are used (e.g. abundance of different species). The aim is then to preserve and to represent the relative positions of the objects and the magnitude of variation between variables in the reduced space. PCA on a correlation matrix is rather used when descriptor variables are measured in different units or on different scales (e.g. different environmental parameters) or when the aim is to display the correlations among (standardized) descriptor variables. The two approaches lead to different principal components and different distances between projected objects in the ordination; hence, the interpretation of the relationships must be made with care (Table 2). Indeed, for correlation matrices, variables are first standardized (i.e. they become independent of their original scales), and so distances between objects are also independent from the scales of the original variables. All variables thus contribute to the same extent to the ordination of objects, regardless of their original variance.

**Table 2:** Interpretation of ordination diagrams

Linear methods (PCA, RDA)					
		
PCA, RDA		RDA			
				Scaling 1	Scaling 2
Samples	Species	ENV	NENV	Focus on sample (rows) distance	Focus on species (columns) correlation
✓				Euclidean distances among samples	–
	✓			–	Linear correlations among species
		✓		Marginal effects of ENV on ordination scores	Correlations among ENV
			✓	Euclidean distance between sample classes	–
✓	✓			Abundance values in species data	
✓		✓		–	Values of ENV in the samples
✓			✓	Membership of samples in the classes	
	✓	✓		Linear correlations between species and ENV	
	✓		✓	Mean species abundance within classes of nominal ENV	
		✓	✓	–	Average of ENV within classes

Unimodal methods (CA, CCA)					
			
CA, CCA		CCA		Focus on sample (rows) distance and Hill's scaling	Focus on species (columns) distances

✓				Turnover distances among samples	χ^2^ distances between samples
	✓			-	χ^2^ distances among species distributions
		✓		Marginal effects of ENV	Correlations among ENV
			✓	Turnover distances between sample classes	χ^2^ distances between sample classes
✓	✓			Relative abundances of the species table	Relative abundances of the species table
✓		✓		–	Values of ENV in the samples
✓			✓	Membership of samples in the classes	
	✓	✓		Weighted averages – the species optima in respect to particular ENV	
	✓		✓	Relative total abundances in the sample classes	
		✓	✓	–	ENV averages within sample classes

The interpretation of ordination diagrams depends on the focus of the study, because sample scores are rescaled as a function of the scaling choice. Approximate relationships between and among the different elements represented in biplots and triplots as species (represented as dots or arrows), samples (dots), environmental variables (ENV; arrows), and nominal (qualitative) environmental variables (NENV; dots). A meaningless interpretation (“–”) happens when the suggested comparison is not optimal because of inappropriate scaling of the ordination scores. Adapted from ter Braak (1994); Leps & Smilauer (1999); ter Braak & Smilauer (2002).

PCA results are generally displayed as a biplot (Jolicoeur & Mosimann, 1960), where the axes correspond to the new system of coordinates, and both samples (dots) and taxa (arrows) are represented (Fig. 1a). The direction of a species arrow indicates the greatest change in abundance, whereas its length may be related to a rate of change. Depending on whether a distance or a correlation biplot is chosen, different interpretations can be made from the ordination diagram (Table 2). The interpretation of the relationships between samples and species differs and is directly affected by the scaling chosen, i.e. whether the analysis mainly focuses on intersample relationships (scaling 1) or interspecies correlations (scaling 2). For instance, in scaling 1, the distances between objects are an approximation of their Euclidean distances in the multidimensional space, but this approximation is not valid if scaling 2 is chosen (Table 2). Projecting an object at a right angle on a species arrow in the ordination diagram approximates the position of the object along that species descriptor. The length of the species descriptor indicates its contribution to the formation of the ordination space. For correlation biplots, the length of the orthogonal projection of a species arrow on the axes approximates its SD on the respective axes. Angles between species arrows reflect their correlations, e.g. putative interactions between species (scaling 2). An erroneous interpretation of the biplot would be to use the proximity of an object point and the tip of a species arrow to deduce a relationship between them. Indeed, only right-angle projections of samples onto species arrows are correct to derive approximated species abundance in the samples.

PCA should generally be used when the objects (sites or samples) cover very short gradients, i.e. when the same species are mostly identified everywhere in the study area (i.e., when samples mostly differ in species abundances), and when species linearly respond to environmental gradients. Because those conditions are often not met in ecological studies, other multivariate approaches have been progressively preferred over PCA (as also suggested by Table 1) such as correspondence analysis or multidimensional scaling.

PCA is successful when most of the variance is accounted for by the largest (generally the first two or three) components. The amount of variance accounted for by each principal component is given by its ‘eigenvalue.’ The mathematical description of eigenvalue calculation steps goes beyond the aim of the present review but can be found in most linear algebra manuals. Eigenvalues derived from a PCA are generally considered to be significant when their values are larger than the average of all eigenvalues (Legendre & Legendre, 1998). The cumulative percentage of variance accounted for by the largest components indicates how much proportion of the total variance is depicted by the actual ordination. High absolute correlation values between the synthetic variables (principal components) and the original variables are useful to identify which variables mainly contribute to the variation in the data set, and this is referred to as the *loading* of the variables on a given axis. However, because the synthetic and original variables are linearly correlated (i.e. they are not independent), standard tests to determine the statistical significance of the correlations between them cannot be used.

#### Principal coordinate analysis (PCoA)

The technique is more rarely used by microbial ecologists (Table 1), despite its usefulness at reducing and representing patterns present in distance matrices displaying dissimilarities among objects (Gower, 1966). Its objectives are very similar to those of PCA in that it uses a linear (Euclidean) mapping of the distance or dissimilarities between objects onto the ordination space (i.e. projection in a Cartesian space), and the algorithm attempts to explain most of the variance in the original data set. In microbial ecology, PCoA has been used, for instance, to test whether virulence profiles (i.e. presence or absence of specific genes) arising from pathogenic strains could differentiate either healthy or contaminated hosts (Chapman *et al*., 2006), or to determine whether PCoA axes could separate groups of *Staphylococcus aureus* isolates into bovine and human hosts when genetic relationships among them had been established by random amplified polymorphic DNA-PCR analysis (Reinoso *et al*., 2004).

As opposed to PCA, PCoA works with any dissimilarity measure and so specific association coefficients that better deal with the problem of the presence of many double zeros in data sets can be surmounted. Moreover, PCoA does not provide a direct link between the components and the original variables and so the interpretation of variable contribution may be more difficult. This is because PCoA components, instead of being linear combinations of the original variables as in PCA, are complex functions of the original variables depending on the selected dissimilarity measure. Besides, the non-Euclidean nature of some distance measures does not allow for a full representation of the extracted variation into a Euclidean ordination space. In that case, the non-Euclidean variation cannot be represented and the percent of total variance cannot be computed with exactness. The choice of the dissimilarity measure is thus of great importance, and subsequent transformation of the data to correct for negative eigenvalues is sometimes necessary (see Legendre & Legendre, 1998, section 9.2.4. for how to correct for such negative eigenvalues).

Objects are represented as points in the ordination space. Eigenvalues are also used here to measure how much variance is accounted for by the largest synthetic variables on each PCoA synthetic axis. Although there is no direct, linear relationship between the components and the original variables, it is still possible to correlate object scores on the main axis (or axes) with the original variables to assess their contribution to the ordination.

#### Correspondence analysis (CA)

A basic question that ecologists may want to address when facing a multidimensional table of sites (or samples) by species is whether certain species occur at specific sites, as a measure of their ecological preferences. CA has generally been used in microbial ecology to determine whether patterns in microbial OTU distribution could reflect differentiation in community composition as a function of seasons, geographic origin, or habitat structure (Olapade *et al*., 2005; Edwards *et al*., 2006a, b; Kent *et al*., 2007). The overall aim of the method is to compare the correspondence between samples and species from a table of counted data (or any dimensionally homogenous table) and to represent it in a reduced ordination space (Hill, 1974). Noticeably, instead of maximizing the amount of variance explained by the ordination, CA maximizes the correspondence between species scores and sample scores. Several algorithms exist and the most commonly described one is *reciprocal averaging*, which consists of (1) assigning arbitrary numbers to all species in the table (these are the initial species scores), (2) for each sample, a sample score is then determined as a weighted average of all species scores (this thus takes into account the abundance of each species at the site and the previously determined species scores), (3) for each species, a new species score is then calculated as the weighted average of all the sample scores, (4) both species scores and sample scores are standardized again to obtain a mean of zero and a SD of one, and (5) steps two to four are repeated until species and site scores converge towards stable solutions in successive iterations (Hill, 1974). The overall table variance (inertia) based on χ^2^ distances is decomposed into successive components that are uncorrelated to each other, as in the PCA or PCoA procedures. For each axis, the overall correspondence between species scores and sample scores is summarized by an eigenvalue, and the latter is thus equivalent to a correlation coefficient between species scores and sample scores (Gauch, 1982).

The technique is popular among ecologists because CA is particularly recommended when species display unimodal (bell shaped or Gaussian) relationships with environmental gradients (ter Braak, 1985), as it happens when a species favors specific values of a given environmental variable, which is revealed by a peak of abundance or presence when the optimal conditions are met (this can be visualized by plotting species abundance against the environmental parameter). The unimodal model that supports the concept of ecological niches has also been shown to be of the right order of complexity for the ordination of most ecological data (ter Braak & Prentice, 1988). Although examples of unimodal distributions along variables or environmental gradients exist with macroorganisms (ter Braak, 1985), the shape of the distribution of the abundance of microbial species along environmental parameters or gradients has not been extensively investigated (but see Ramette & Tiedje, 2007a, b). This may arise from the fact that, in microbial surveys, environmental sampling is mostly performed blindly in relation to environmental heterogeneity, and the abundance of target species is generally determined without systematically analyzing associated environmental parameters. Finally, another important feature of CA for microbial ecologists is that the reciprocal averaging algorithm disregards species double absences because the relationships between rows and columns of the table are quantified using the χ^2^ coefficient that excludes double absences (Legendre & Legendre, 1998).

**Fig. 2:**
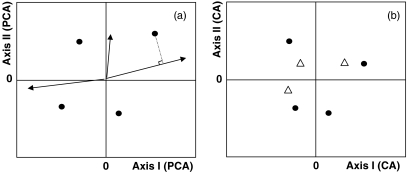
Ordination diagrams in two dimensions. (a) In a PCA biplot representation, samples are represented by dots and species by arrows. The arrows point in the direction of maximal variation in the species abundances, and their lengths are proportional to their maximal rate of change. Long arrows correspond to species contributing more to the data set variation. Right-angle projection of a sample dot on a species arrow gives approximate species abundance in the sample. (b) In a CA joint plot representation focusing on species distance, both samples and species are depicted as dots. Species dots correspond to the center of gravity (inertia) of the samples where they mostly occur. Distances between sample and species points give an indication of the probability of species composition in samples (see Table 2 for more details about diagram interpretation).

Both samples and taxa are often jointly depicted in the ordination space (i.e. joint plot; Fig. 2b), where the center of inertia (centroid) of their scores corresponds to the zero for all axes. Depending on the choice of the scaling type, either the ordination of rows (samples) or the columns (species) is meaningful, and can be interpreted as an approximation of the χ^2^ distances between samples or species, respectively (see Table 2 for more details about interpretation). Sample points that are close to each other are similar with regard to the pattern of relative frequencies across species. It is important to remember that in such joint plots, either distances between sample points or distances between species points can be interpreted, but not the distances between sample and species points. Indeed, these distances are not simple Euclidean distances computed from the relative row or column frequencies, but rather they are weighted distances. The proximity between sample and species points in the plot can thus be understood as a probability of species occurrence or of a high abundance in the samples in the vicinity of a species point.

In scaling 2 (i.e. focus on species), species points found at the center of the ordination space should be carefully checked with the raw data to clarify whether the species ordination really corresponds to the optimal abundance or occurrence of the species, or whether the species is just badly represented by the main axes, as it is the case when other axes are more appropriate to represent the species. Rare species contribute little to the total table inertia (i.e. they only play a minor role in the overall table variance) and are hence positioned at the edges of the plot, next to the site(s) where they occur. In general, only the species points found away from the ordination center and not close to the edges of the ordination have more chances to be related to the ordination axes, i.e. to contribute to the overall variance (Legendre & Legendre, 1998).

When the species composition of the sites progressively changes along the environmental gradient, sample positions may appear in the ordination plot as nonlinear configurations called ‘arch’ (Gauch, 1982) (or ‘horseshoe’ in the case of PCA), which may impair further ecological interpretation. In CA, the arch effect may be mathematically produced as a side-effect of the CA procedure that tries to obtain axes that both maximally separate species and that are uncorrelated to each other (ter Braak, 1987): when the first axis suffices to correctly order the sites and species, a second axis (uncorrelated with the former) can be obtained by folding the first axis in the middle and bringing its extremities together, thus resulting in an arch configuration. Further axes can be obtained by further dividing and folding the first axis into segments (Legendre & Legendre, 1998). To remove the arch effect in CA, a mathematical procedure, *detrending*, is used to flatten the distribution of the sites along the first CA axis without changing their ordination on that axis. The approach is then designated as detrended correspondence analysis (DCA). The review of different detrending algorithms such as using segments or polynomials goes beyond the scope of this review, but more information can be obtained in (ter Braak & Prentice, 1988; Legendre & Legendre, 1998). Some authors have also argued that the arch effect may not be an artifact but an expected feature of the analysis, especially when species turnover is high along environmental gradients (James & McCulloch, 1990). In that case, if the samples are meaningfully positioned along the arch, the ordination should be accepted as a valid result.

#### Nonmetric multidimensional scaling (NMDS)

NMDS is generally efficient at identifying underlying gradients and at representing relationships based on various types of distance measures. Not surprisingly, NMDS has found an increasing number of applications in microbial ecology (Table 1). The technique has been generally applied to identify patterns among multiple samples that were subjected to molecular fingerprinting techniques. For instance, NMDS was used to analyze and to compare the reproducibility of various fingerprinting techniques such as ribosomal internal spacer analysis (RISA), terminal fragment length polymorphism (T-RFLP), and denaturing gradient gel electrophoresis (DGGE) between different laboratories when applied to samples chosen from a salinity gradient (Casamayor *et al*., 2002). NMDS was also used to compare diversity patterns of microbial communities (as determined by length heterogeneity-PCR) from samples undergoing different land management practices (Mills *et al*., 2006). Another example is the analysis of the bacterioplankton communities of four shallow eutrophic lakes that differed in nutrient load and food web structure using DGGE profiling, so as to determine the specificity of community signatures in each lake (Van der Gucht *et al*., 2005).

The NMDS algorithm ranks distances between objects, and uses these ranks to map the objects nonlinearly onto a simplified, two-dimensional ordination space so as to preserve their ranked differences, and not the original distances (Shepard, 1966). The procedure works as follows: the objects are first placed randomly in the ordination space (the desired number of dimensions has to be defined *a priori*), and their distances in this initial configuration are compared by monotonic regression with the distances in the original data matrix based on a stress function (values between 0 and 1). The latter indicates how different the ranks on the ordination configuration are from the ranks in the original distance matrix. Several iterations of the NMDS procedure are generally implemented so as to obtain the lowest stress value possible (i.e. the best goodness of fit) based on different random initial positions of the objects in the ordination space. For sample-by-species tables, simulations have shown that before applying NMDS, a standardization of each species by its maximum abundance, followed by the computation of distances between samples based on the Steinhaus or Kulczinski similarity coefficients yielded informative ordination results (Legendre & Legendre, 1998, p. 449).

In NMDS ordination, the proximity between objects corresponds to their similarity, but the ordination distances do not correspond to the original distances among objects. Because NMDS preserves the order of objects, NMDS ordination axes can be freely rescaled, rotated, or inverted, as needed for a better visualization or interpretation. Because of the iterative procedure, NMDS is more computer intensive than eigenanalyses such as PCoA, PCA, or CA. However, constant improvement in computing power makes this limitation less of a problem for small- to medium-sized matrices.

### Testing for significant differences between groups

In addition to representing objects in an ordination plot or as clusters of similar objects, another objective may be to test whether differences between groups of objects (rows) in a multivariate table are significantly different based on the set of their attributes (columns), i.e. to test whether similarities within groups are higher than those between groups. Here, nonparametric multivariate anova (npmanova) and analysis of similarities (anosim), which are commonly found in standard statistical packages, are briefly reviewed. It is also possible to use canonical analyses (‘Testing for significant differences between groups’) to test for significant differences between groups of objects. These statistical tests, however, must not be used to assess the statistical difference among groups that were derived from a previous cluster analysis on the same variables because, under those conditions, the two approaches would not be independent from each other. Indeed, the groups derived from cluster analysis (which are themselves made to fit the data) would then be used for testing the null hypothesis that there is no difference among the groups. This hypothesis would then not be independent of the data used to test it, and would nearly always produce significant differences between the groups even if it is not the case (Legendre & Legendre, 1998).

#### npmanova

The method can be used to test for significant differences between the means of two or more groups of multivariate, quantitative data (Anderson, 2001). The null hypothesis of equality of means is tested based on Wilks' Λ (lambda) statistic, which replaces the *F*-test normally used in univariate anova. When only two groups are compared, Hotelling's *T*^2^ test is more appropriate. The latter test can also be used, as a *post hoc* test, to assess the significance of pairwise comparisons statistically between groups, following an overall significant Wilks' test. Significance is generally computed by permutation of group membership, with several thousand replicates, alleviating concerns about multinormality of the data. Because multiple pairwise comparisons are made, the significance level of the pairwise Hotelling's tests needs, however, to be corrected. With the Bonferroni correction, for instance, the *P*-value usually chosen for significant differences between groups (i.e. 0.05) is replaced by a smaller *P*-value calculated by dividing the original *P*-value by the total number of pairwise comparisons that are performed. For instance, for 10 pairwise comparisons, the corrected *P*-value becomes 0.005. This correction is often judged to be rather conservative as it leads to significance for fewer pairwise comparisons (Legendre & Legendre, 1998).

#### anosim

This nonparametric procedure tests for significant difference between two or more groups, based on any distance measure (Clarke, 1993). It compares the ranks of distances between groups with ranks of distances within groups. The means of those two types of ranks are compared, and the resulting *R* test statistic measures whether separation of community structure is found (*R*=1), or whether no separation occurs (*R*=0). *R* values >0.75 are commonly interpreted as well separated, *R*>0.5 as separated, but overlapping, and *R*<0.25 as barely separable (Clarke & Gorley, 2001). The test makes fewer assumptions than manova because it is based on the ranks of distances, and it is often used for sample-by-species tables, where groups of samples are compared. All groups should have comparable within-group dispersion to avoid finding falsely significant results (Legendre & Legendre, 1998).

Applications in microbial ecology include testing for spatial differences, temporal changes, or environmental impacts on microbial assemblages. For instance, Kent *et al*. (2007) determined whether bacterial communities from the same lake were more similar in composition to each other than to communities in different lakes. The bacterial composition and diversity of samples from different geographic origins, habitats, and avian hosts were also compared using anosim based on a length heterogeneity (LH)-PCR (Bisson *et al*., 2007). Another example is the application of anosim to terminal restriction fragment length polymorphism (T-RFLP)-generated data to determine the impact of B and NaCl on soil microbial community structure in the wheat rhizosphere (Nelson & Mele, 2007).

## Environmental interpretation

Exploratory analyses may reveal the existence of clusters or groups of objects in a data set. When a supplementary table or matrix of environmental variables is available for those objects, it is then possible to examine whether the observed patterns are related to environmental gradients. Typical objectives may be, for instance, to reveal the existence of a relationship between community structure and habitat heterogeneity, between community structure and spatial distance, or to identify the main variables affecting bacterial communities when a large set of environmental variables has been conjointly collected.

The significance of the relationships between species patterns and environmental variables can generally be assessed by permutation techniques such as Monte Carlo permutation tests, which infer statistical properties from the data themselves. The order of data (generally the rows of one matrix) is permuted and the relationships between the observed patterns and environmental variables can be assessed for randomness. This approach is particularly suitable when variables do not follow a normal distribution (which is often the case with environmental or ecological data), as generally required by traditional statistical procedures (Legendre & Legendre, 1998).

### Indirect gradient analyses

Ordination axes or clusters can be interpreted based on additional environmental variables (i.e. variables not used in the ordination or cluster analysis) that provide ecological knowledge about the studied sites or species ecological characteristics. When using exploratory ordination approaches on a sample-by-species table, samples are displayed along the axes of main variation in species composition. These axes are thus constructed without reference to environmental characteristics, but they can be hypothesized to represent underlying environmental gradients (e.g. environmental parameters, spatial or temporal variables, chemical gradients), which need to be subsequently identified. Such an approach is designated as ‘indirect,’ because synthetic variables (i.e. the axes) are first constructed and thereafter related to environmental variation. For instance, the scores of the objects on PCA or CA main components (axes) can be further related by standard statistical procedures (e.g. anova, regression analysis) to environmental variables. Likewise, in PCoA or NMDS, it is possible to statistically compare the ranks obtained by the objects on each axis and the ranks of those objects on additional environmental variables, using Spearman's rank correlation coefficients (Legendre & Legendre, 1998).

A convenient method of interpretation is to represent the additional environmental variables as fitted arrows directly on the ordination diagram. These variables are added to the existing ordination by linear regression of their values onto the existing ordination axes. This procedure is implemented in various statistical packages (e.g. canoco, R). Hence, it is possible to assess the direction and magnitude of the most rapid change in the environmental variables and to determine whether they correspond to the observed patterns among objects (Oksanen, 2007). In cluster analysis, the magnitude of the absolute correlation value between an ordered clustering solution and environmental variables may also provide clues about putative environmental causes for the observed discontinuities in the data set.

Another convenient way of displaying additional information to help interpret the ordination is to use site symbols whose sizes are proportional to the values of the additional variable. Hence, one can visually assess whether the ordination of objects (samples, sites) matches specific trends in the additional variable. This strategy was, for instance, used on NMDS ordination plots inferred from DGGE profiles on which the values of five additional environmental variables were individually mapped as proportional circles in order to identify the main environmental factors related to the bacterial community structure in four freshwater lakes (Van der Gucht *et al*., 2005).

### Direct gradient analyses (constrained analyses)

In constrained (canonical) ordination analyses, only the variation in the species table that can be explained by the environmental variables is displayed and analyzed, and not all the variation in the species table. Gradients are supposed to be known and represented by the measured variables or their combinations, while species abundance or occurrence is considered to be a response to those gradients. Constrained ordinations are mostly based on multivariate linear models relating principal axes to the observed environmental variables, and the different techniques depend on data types (matrix or table), and on the hypothesis underlying species distribution in the gradients (i.e. linear or unimodal). Their aim is to find the best mathematical relationships between species composition and the measured environmental variables, and to assess whether, statistically, such a relationship could have been produced due to chance alone using permutation tests. The resulting ordination diagrams display samples, species, and environmental variables so that ‘fitted species × samples’ and ‘species × environment’ relationships can be derived as easily as possible from angles between arrows or distances between points and arrows (Table 2).

#### Redundancy analysis (RDA)

In microbial ecology, RDA has been applied, for instance, to test whether the occurrence of biocontrol bacteria with specific carbon source utilization profiles was related to their origin from different root samples (Folman *et al*., 2003), to determine which environmental factors were the most significant to explain variation in microbial community composition in undisturbed native prairies and cropped agricultural field (McKinley *et al*., 2005), to examine the effects of sampling locations (longitude, latitude, altitude) on genetic diversity of plant pathogenic bacteria (Kolliker *et al*., 2006), or to assess the influence of season, farm management, and soil chemical, physical, and biological properties on nitrogen fluxes and bacterial community structure (Cookson *et al*., 2006).

This method can be considered as an extension of PCA in which the main axes (components) are constrained to be linear combinations of the environmental variables (Rao, 1964). Two tables are then necessary: one for the species data (‘dependent’ variables) and one for the environmental variables (‘independent’ variables). Multiple linear regressions are used to ‘explain’ variation between independent and dependent variables, and these calculations are performed within the iterative procedure to find the best ordination of the objects. The interest of such an approach is to represent not only the main patterns of species variation as much as they can be explained by the measured environmental variables but also to display correlation coefficients between each species and each environmental variable in the data set.

When the data set consists of a matrix of distances between objects, distance-based RDA (db-RDA; Legendre & Anderson, 1999) can be applied to determine how well additional environmental parameters can explain the variation among objects in the matrix. The technique first applies a PCoA on the distance matrix to convert it back to a rectangular table containing rows of objects by columns of PCoA coordinates. Those new, uncorrelated coordinates thus correspond to synthetic ‘species’ variables that are then related to additional environmental parameters using a classical RDA. For instance, db-RDA was successfully used to determine how the variation in matrices of genomic distances among environmental strains could be explained by factors such as soil parameters, host plant species, and spatial scale, each factor being taken alone or in combination (Ramette & Tiedje, 2007b).

Most software outputs provide the total variation in species composition as explained by the environmental axes, the cumulative percentage of variance of the species–environment relationship, and the overall statistical significance of the relationships between the species and environmental tables. RDA can be represented by a triplot of samples (dots), species (arrows), and environmental variables (arrows for quantitative variables and dots for each level of qualitative or nominal variables), or by any combinations thereof (i.e. biplots) (ter Braak, 1994). Depending on the scaling chosen, i.e. whether the analysis mainly focuses on intersample relationships or interspecies correlations, the interpretation of the relationships between samples, species, and environmental variables differs (Table 2).

#### Canonical correspondence analysis (CCA)

The approach is very similar to that of RDA, except that CCA is based on unimodal species–environment relationships whereas RDA is based on linear models (ter Braak, 1986). CCA can be considered as the constrained form of CA in which the axes are linear combinations of the environmental variables. CCA uses the unimodal model to model species response to the environmental variation as a mathematical simplification to enable the estimation of a large number of parameters and the identification of a small number of ordination axes. This species model seems, however, to be robust even when some species display bimodal responses, unequal ranges, or unequal maxima along environmental gradients, and the technique is thus considered to be the method of choice by many ecologists (ter Braak & Smilauer, 2002). It is therefore particularly adapted for the environmental interpretation of tables of abundance and occurrence of species, and accommodates well the absence of species at certain sites in the data set. CCA is sensitive to rare species that occur in species-poor samples, and down-weighting of such species help reduce the problem (Legendre & Legendre, 1998). Software outputs are very similar to those of RDA and as for RDA, triplot and biplot representations and interpretation depend on the choice of the scaling type (Table 2). The same interpretation of the relationships between sample and species points is found in CA and CCA. Right-angle projection of these points on the environmental arrows leads to the correct approximation of the ranking of the points along environmental variables.

CCA has been used in an increasing number of publications dealing with microbial assemblages in marine and soil ecosystems. Typical questions that are addressed concern the identification of environmental factors that influence the diversity of bacterial assemblages among large sets of candidate environmental parameters measured for the same samples, when the diversity is determined by culture-independent, genetic fingerprinting techniques such as automated ribosomal intergenic spacer analysis (ARISA) (Yannarell & Triplett, 2005), DGGE (Salles *et al*., 2004; Sapp *et al*., 2007), or T-RFLP (Córdova-Kreylos *et al*., 2006; Klaus *et al*., 2007). Another interest in the technique comes from the possibility of determining the specific species or OTUs that respond to particular environmental variables, and as such that can be identified as candidate *indicator species*. Those species can then be subjected to further experiments so as to confirm their status of indicator species. For instance, the relationships, as determined by CCA, between bacterial community composition and 11 environmental variables for 30 lakes in Wisconsin, revealed that patterns in bacterial communities were best explained by regional- and landscape-level factors, as well as by specific seasons, pH, and water clarity (Yannarell & Triplett, 2005). CCA was also successfully used to demonstrate that former land use management affected the composition of the targeted soil microbial community (*Burkholderia*) to a larger extent than did plant species (Salles *et al*., 2004). Another interesting example in the marine ecosystem is the study of the interactions between various abiotic parameters and phytoplankton community data (biotic parameter) to explain bacterioplankton dynamics in the North Sea and the subsequent identification of the bacterial phylotypes responding more specifically to the factors (Sapp *et al*., 2007). Another example of using CCA to identify some microbial communities as pollution indicators can be found in (Córdova-Kreylos *et al*., 2006).

#### Partial ordination, variation partitioning

When the effects of a particular environmental variable need to be tested after elimination of possible effects due to other (environmental) variables, partial ordination may be used (e.g. partial CCA, partial RDA). Such an approach is also referred to as ‘partialling out’ or ‘controlling for’ the effects of specific variables, which are specified as covariables in the constrained analysis. For instance, in a study dealing with the effects of environmental and pollutant variables on microbial communities, Córdova-Kreylos *et al*. (2006) observed that variation in microbial communities was more due to spatial variation than to pollutants. The use of partial CCA to account for spatial variation in the biological data set revealed that metals had a greater effect on microbial community composition than organic pollutants.

**Fig. 3:**
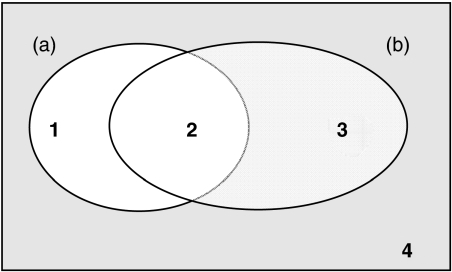
Partitioning biological variation into the effects of two factors. The large rectangle represents the total variation in the biological data table, which is partitioned among two sets of explanatory variables (a, b). Fraction 4 shows the unexplained part of the biological variation. Fractions 1 and 3 are obtained by partial constrained ordination or partial regression, and can be tested for significance. For instance, fraction 1 corresponds to the amount of biological variation that can be exclusively explained by (a) effects when (b) effects are taken into consideration (i.e., when b is considered as a covariable). Fraction 2 [i.e., variation indifferently attributed to (a) and (b) or a covariation of (a) and (b)] is obtained by subtracting fractions 1 and 3 from the total explained variance, and cannot be tested for statistical significance.

This idea of controlling for the effects of specific variables can be extended to evaluate the effects of all the different sets (factors) of environmental variables present in a study so as to determine the relative contribution (amount of variation explained) and significance of each variable set on the total biological variance. The so-called *variation partitioning* procedure (Borcard *et al*., 1992) partitions the total variance of the species table into the respective contribution of each set of environmental variables and into their covariations using both standard and partial constrained ordinations (Fig. 3). Two methods have traditionally been used to partition the variation of community composition data, i.e. canonical partitioning and regression on distance matrices based on Mantel tests (Legendre & Legendre, 1998). The canonical approach has been shown to be more appropriate to partition the β diversity correctly among sites and to test hypotheses about the origin and maintenance of its variation (Legendre *et al*., 2005).

Applications of variation partitioning in microbial ecology include, for instance, the study by Ramette & Tiedje (2007b), which applied the technique in the context of RDA to disentangle the effects of space, environmental soil parameters, and plant species on *Burkholderia* community abundance and diversity. By quantifying the amount of biological variation that is left unexplained when all environmental variables had been considered, the study suggested that much less of the biological variation could be predicted at the intraspecific level compared with higher taxonomic levels. Another interesting example is the study of seasonal changes in bacterial community composition in shallow eutrophic lakes, in which top-down regulation (grazers) of bacterial community composition was examined after accounting for bottom-up regulation (resources) (Muylaert *et al*., 2002).

#### Linear discriminant analysis (LDA)

When groups or clusters of objects have been obtained by exploratory analyses for instance, LDA can be used to identify linear combinations of additional environmental variables that best discriminate those groups. In that respect, LDA can be seen as an extension of manova for two or more groups, in which environmental variables that specifically explain the grouping of objects are identified. Another application consists of assigning new objects to previously defined groups for prediction or classification purposes based on the calculated discriminant function. For instance, Fuhrman *et al*. (2006) used the technique to evidence the existence of repeatable temporal patterns in the community composition of marine bacterioplankton over 4.5 years.

The technique is mostly recommended for multinormal data for which attribute data are linearly related and for which variances and covariances of the variables are good summary statistics. A visual representation of LDA can be performed, and in the resulting ordination, the axes are then the discriminant functions. The distances between objects, which correspond to Mahalanobis distances that take into account the correlations among descriptors (Mahalanobis, 1936), are independent of the scale of measurement of the various descriptors and are mostly used to compare groups of sites or objects with each other (Legendre & Legendre, 1998).

#### Selection of variables in regression models

In the previous constrained methods where linear combinations of environmental (explanatory) variables are used, the inclusion of too many explanatory variables to describe species distribution may lead to difficult ecological interpretations and to lower predictability of the models, due to intercorrelations among the explanatory variables (i.e. multicollinearity). Multicollinearity has the effects of inflating the variance of the regression coefficients in the models, leading to reduced precision in the prediction of the response variables (Legendre & Legendre, 1998). In order to only include in the model the environmental variables that mostly and significantly contribute to the variation of the species table, automatic selection procedures (forward selection, backward elimination, or stepwise selection) are often used. The selection depends on whether the partial correlation coefficients of the variables fall below a given significance level, the latter being generally assessed by Monte Carlo permutation tests.

In forward selection, the construction of the regression model starts with the variable that explains the most variation in the dependent variables (generally the species table). What remains of the biological variation to explain after fitting the first environmental variable (i.e. of the residual variation) is then used to choose the second environmental variable. The process of selection goes on until no more variables significantly explain the residual variation. In backward elimination, the construction of the regression model starts with all environmental variables and the least significant ones are excluded from the model, one at a time until a group of only ‘significant’ variables is obtained. To take advantage of the two approaches, stepwise regression mixes forward selection with backward elimination by performing a forward selection, but excluding the variables that no longer become significant after the introduction of new variables into the regression model.

Despite the clear advantages of these variable selection strategies, most authors still caution that researchers should not blindly rely on automatic selection procedures to choose the relevant environmental variables in regression models because ecologically irrelevant models may also be obtained, or other variable combinations could also yield better models to explain species variation (Legendre & Legendre, 1998). Noticeably, the three selection strategies do not necessarily yield the same set of significant environmental variables, because they may be seen as heuristic methods to identify a significant model when all possible combinations of significant models are not possible to evaluate computationally. Another approach is thus to combine variables into biologically or environmentally meaningful sets, instead of relying on automatic selection procedures, and then to examine all possible regression models based on the reduced number of variable sets (James & McCulloch, 1990). For instance, before applying variation partitioning to different groups of variables representing spatial scales (15 variables), host species (four variables), and soil parameters (10 variables), Ramette & Tiedje (2007b) applied forward selection within each group to determine the variables significantly explaining the variation of microbial diversity and abundance at different taxonomic levels.

#### Mantel test

This test is appropriate to compare two matrices that were calculated for the same objects but that are based on two independent data sets (e.g. a species dissimilarity matrix and an environmental dissimilarity matrix for the same samples) (Mantel, 1967). It calculates the correlation coefficient between corresponding positions in the two matrices, and assesses its significance based on permutations of the objects in one of the matrices. In microbial ecology, the Mantel test has become popular especially for testing the relationships between molecular and geographic distance matrixes for a same set of organisms or to relate community diversity to environmental heterogeneity (e.g., Parker & Spoerke, 1998; Cho & Tiedje, 2000; Horner-Devine *et al*., 2004; Scortichini *et al*., 2006).

Another interesting application, called a goodness-of-fit Mantel test, corresponds to the case where one matrix is recoded to represent ecological hypotheses to be tested on the other matrix (Legendre & Legendre, 1998). For instance, if a matrix of molecular data is available for a set of strains and their habitat of origin is known, it is possible to determine whether the genetic distances are related to habitat type using the (goodness-of-fit) Mantel test. The matrix representing the ecological hypotheses should then consist of a series of 1 and 0 for isolates found in the same or different habitats, respectively. The Mantel test can thus determine whether the posited habitat distribution can significantly explain the structure of the molecular matrix. This test cannot be used, however, to test a hypothesis matrix that would be based on the results of a cluster analysis, for instance. Indeed, as indicated in ‘Testing for significant differences between groups,’ there would be a lack of independence between the hypothesis being tested and the data used to test the hypothesis.

Note that the Mantel test is also used to compute Mantel correlograms, which are often found in biogeographical studies (e.g. Mantel correlograms are usually used to detect spatial structure in species assemblages based on grouping of the response data into specific spatial distance classes). Mantel tests are then applied to each group in order to detect significant correlations at a given scale, i.e. the scales at which the data are autocorrelated (Legendre & Legendre, 1998).

## Practical considerations

### Choice of an ordination method (Fig. 4)

Linear methods such as multiple regression, LDA, PCA, or RDA are generally meant to be applied to continuous data. Their use is thus sometimes limited in Ecology where species generally display nonlinear, nonmonotone responses to environmental variables (ter Braak & Prentice, 1988; Legendre & Legendre, 1998). Different approaches can be undertaken to choose the most appropriate ecological model. Plot of species abundances along ordination axes or explanatory variables (also called coenocline) may help visualize whether species responses are linear or unimodal (ter Braak & Smilauer, 2002). Besides, the choice of linear (PCA, RDA) or unimodal (CA, CCA) species response models can be made on the basis of whether the underlying gradient length is short or long, respectively. Gradient length, as measured in SD units along the first ordination axis, can be estimated by DCA for unconstrained ordination and by detrended CCA (DCCA) for constrained ordination in, e.g. the software canoco (ter Braak & Smilauer, 2002). It is recommended to use linear methods when the gradient length is <3 SD, unimodal methods when it is >4 SD, and any method for intermediate gradient lengths (ter Braak & Smilauer, 2002).

**Fig. 4:**
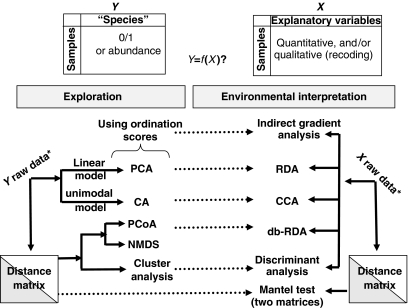
Relationships between numerical methods. Exploratory tools such as PCA, CA, PCoA, NMDS, or cluster analysis can be applied to a sample-by-species table to extract the main patterns of variation, to identify groups or clusters of samples, or specific species interactions. Sample scores on the main axes of variation can be related to variation in environmental variables using indirect gradient analyses. When a constrained analysis is desired (i.e. direct gradient analysis), RDA, db-RDA, CCA, or linear discriminant analysis can be used as extensions of the unconstrained methods. Mantel tests are appropriate to test the significance of the correlation between two distance matrices (e.g. one based on species data and the other on environmental variables). Raw data may be transformed, normalised or standardised as appropriate before analysis.

Data type is also another important criterion. To represent absolute abundance values, linear-based methods (PCA, RDA), which produce weighted summations, are appropriate, whereas unimodal techniques (CCA, CA) are rather used to model relative abundances (because species scores are weighted averages of the samples scores, and vice versa), i.e. they model the dissimilarities between samples (β diversity). They also accommodate well the presence of many zeros in the species table, in contrast to linear-based methods for which double zeros lead to inadequate estimates of sample distances.

Cluster analysis is the method of choice when relationships between objects are expected to be discontinuous and where defined categories or groups of objects are expected. On the contrary, ordination would be more useful when the variation between objects is posited to be continuous. Although NMDS is more computer intensive than PCoA, it is generally better at compressing the distance relationships among objects into a few dimensions. This is because NMDS can always lead to a Euclidean representation even for non-Euclidean embeddable distances (Legendre & Legendre, 1998). NMDS and PCoA can be compared using Shepard diagrams to decide which technique better represents the original distances.

If one assumes that species do not have a linear response to environmental gradients, NMDS is more appropriate than PCA. CA may also be an alternative to PCA when many zeros populate the data set and one strong gradient is present. With long ecological gradients, however, CA may produce the arch effect that can be corrected for using DCA. In terms of the underlying species model, the main difference between DCA and NMDS is that the former is based on a specific model of species distributions (unimodal model), while NMDS is not. Thus, DCA may be favored by ecologists who assume that the niche theory better fits their data set, while NMDS may be a method of choice if species composition is determined by factors other than position along a gradient (for instance if the habitat is known to be fragmented).

In constrained and unconstrained ordinations, all species are posited to react to different extents to the same composite gradients of environmental variables, whereas in a multiple regression approach, a different gradient could be modeled for each species separately. Because most species do not respond linearly to environmental gradients, fitting nonlinear models to individual species may be difficult, especially when dealing with a huge data set. Constrained ordinations thus provide a good summary of species–environment relationships and can be very successful in ecological data analysis (ter Braak & Prentice, 1988). It is also useful to note that RDA is very similar to manova, but in contrast to the latter, RDA allows the consideration of any number of species (columns) (Legendre & Legendre, 1998).

Constrained and unconstrained (exploratory) methods should be used in parallel (Fig. 4) because, with the former, only the biological variation that can be explained by the available environmental variables is represented on the main axes, whereas with unconstrained methods, the highest amount of variance is extracted from the biological data alone and represented on a few axes. If the constrained and unconstrained approaches yield the same ordination of the samples (objects), it thus means that the measured environmental variables explain most of the biological variation. In order to compare the results of different ordinations, a useful technique is *Procrustes* analysis (Gower, 1975), which estimates the concordance of scores in two ordinations after rotating, translating, and dilating them in order to obtain the best fit. A permutation procedure can also be used to test for the significance of the concordance between ordinations or matrices (Peres-Neto & Jackson, 2001).

**Fig. 5:**
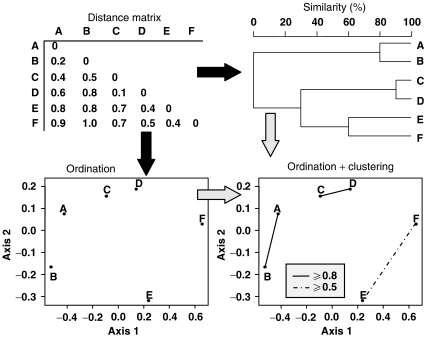
Combination of ordination and cluster analysis. On a same distance matrix, NMDS or PCOA can be applied to represent the major axes of variation among objects in a two-dimensional space. The superimposition of the results of cluster analysis (primary connections) onto the ordination diagram can help identify the structure in the data set as discontinuities (clusters) into a continuous space (ordination). Adapted from Legendre & Legendre (1998).

Cluster analysis and ordination techniques can be combined to provide powerful visualization tools. For instance, hierarchical clustering can help obtain a better interpretation of ordination diagrams (Fig. 5). Because ordination diagrams represent most of the data set variation into a dimensionally reduced space, some relationships among objects can be distorted because only a few projection axes are considered. The addition of linkage results obtained from cluster analysis may help identify objects belonging to the same clusters even if their relative position in the ordination diagram is not ideal (Legendre & Legendre, 1998).

### Ordination and diversity indices

The measurement of diversity is generally performed using indices such as the Shannon or Simpson indices. The latter are often applied to measure different components of the diversity such as α, β, and γ diversity, corresponding to diversity within a particular site or ecosystem, to change in species composition from site to site (i.e. species turnover), and to the diversity at the landscape scale, respectively (Whittaker, 1972). The ordination approach sounds similar, in that variation among samples is compared based on their within-sample composition in species assemblages, and so some of the α and β diversity should be depicted on ordination diagrams. Because diversity indices pool the multispecies information into a single value for each observation, before comparing them, it is not surprising that complex diversity patterns may not be identified sometimes. For instance, Hartman & Widmer (2006) did not find significant changes in soil bacterial communities submitted to various soil managements when using diversity indices, while community structures were shown to have changed using community fingerprinting analysis.

To obtain a consistency between ordination techniques and diversity index measurements, two numerical strategies have been proposed: for species occurrence data, the CA-species richness strategy adapted for data set rich in rare species, and the Nonsymmetric CA – Simpson strategy, which is more appropriate for tables dominated by abundant species (Pelissier *et al*., 2003). These strategies attribute specific weights to the species data so that simple or constrained ordinations of the new species table represent the total inertia as α and β diversity, and would thus be consistent with the measures obtained by common diversity indices.

### Misconceptions about multivariate analyses

It is essential to reiterate that multivariate statistical procedures may suggest causes or factors, but investigators should bear in mind that the synthetic variables, axes, or clusters derived do not necessarily correspond to biological or ecological entities in nature (James & McCulloch, 1990). One should thus not overinterpret the data by relying on unjustified causality, especially in the absence of real experimentation. In theory, it would be necessary to validate the inferences and models made about pattern formation and putative causes by analyzing new data, but this is rarely performed in practice. Moreover, whether the originally collected data are typical of the situation to be described is most of the time not even questioned.

Another common misconception is that multivariate analyses alone can sort out all solutions of complex multivariate studies. Although exploratory analyses may help reveal interesting patterns in data sets, the interpretation and explanation of the observations ultimately rely on the researcher's hypotheses and previous knowledge of the ecological situation. Microbial ecologists themselves need to formulate ecologically sound hypotheses and test them.

## Conclusions

Exciting questions in Ecology typically consist of determining whether community patterns are structured across space or time, of explaining how those patterns can be related to environmental heterogeneity, and of quantifying how much still remains unexplained when all significant, measured variables have been considered. Such questions can now start to be addressed in microbial ecology because numerical tools may help explore and test such ecological hypotheses. These are indeed exciting times because even larger and more complex databases are being created and in parallel, computing power gradually becomes less of an issue. If microbial ecologists want to test numerical methods, develop new ecological theories, or validate existing ones for the microbial case, access to diversity data and above all, to the relevant associated environmental parameters, becomes a central issue. It would thus be of great interest to make such complex data sets publicly available, such as microbial ecological databases, so that microbial diversity can be studied in its environmental context. This would indeed be a step toward making microbial ecology a central discipline in Ecology.

## Statement

Reuse of this article is permitted in accordance with the Creative Commons Deed, Attribution 2.5, which does not permit commercial exploitation.
